# Personality Traits and Producer Behavior: The Influence of Individual Differences in Human Social Foraging

**DOI:** 10.3390/brainsci16020180

**Published:** 2026-01-31

**Authors:** Iván Uribe, Laurent Ávila-Chauvet, Diana Mejía

**Affiliations:** Department of Psychology, Sonora Institute of Technology (ITSON), Ciudad Obregón 85000, Mexico; ivan.uribe233052@potros.itson.edu.mx (I.U.); laurent.ac.ags@gmail.com (L.Á.-C.)

**Keywords:** foraging, big five, metatraits, psychopathy, producer-scrounger

## Abstract

**Background**: During social foraging, individuals typically adopt one of two mutually exclusive strategies: (1) producing, which involves searching for, discovering, and acquiring resources, or (2) scrounging, which entails exploiting resources previously discovered by others. The distribution of these strategies within a group is referred to as the Producer–Scrounger (P-S) Game. Although the influence of personality on the Producer–Scrounger Game has been examined in non-human species through measures of individual differences, few studies have yet explored this relationship in humans. **Objective**: We aimed to examine the association between social foraging strategies and personality traits in human participants, using the Big Five dimensions: openness, conscientiousness, extraversion, agreeableness, and neuroticism, with their higher-order metatraits measured as composite scores: stability and plasticity, and psychopathy traits measured with the Antisocial Process Screening Device (APSD): callous–unemotional, impulsivity, and narcissism. **Methods**: Forty-five participants completed the Guaymas Foraging Task (GFT), designed to simulate a social foraging scenario under two 4 min conditions: one in which the cost of producing was 0 s, and another in which it was 8 s. Participants also completed the Big Five Inventory and the APSD. **Results**: Openness (*p* = 0.018, R^2^ = 0.124), agreeableness (*p* = 0.002, R^2^ = 0.209), extraversion (*p* = 0.019, R^2^ = 0.121), stability (*p* = 0.022, R^2^ = 0.117), and plasticity (*p* = 0.007, R^2^ = 0.160) traits were associated with higher producer’s indexes. However, these correlations emerged only under the low-cost condition. No correlations were found between the producer’s index and psychopathic traits; nonetheless, participants above the APSD’s cutoff score scrounged significantly more, but only in the low-cost condition. **Conclusions**: Individual differences such as personality seem to be correlated with different foraging strategies; nonetheless, the behavioral expression of these traits seems to diminish when the environment is not favorable for their preferred strategy.

## 1. Introduction

Foraging refers to the search for resources and represents the most fundamental aspect of animal behavior, as it underlies functions as essential as reproduction, survival, and the development of every living organism [1]. In social foraging situations, where two or more organisms search for resources, the members of a group can choose between two mutually exclusive yet coexisting strategies: (1) producing, where they invest time on the search, discovery, and acquisition of resources, and (2) scrounging, where they look for opportunities to join a previous discovery and exploit the resources found by a producer [2,3]. The distribution of the use of these strategies among the members of a group is referred to as the producer–scrounger (P-S) Game [2].

In 1991, Vickery et al. [4] developed the Rate Maximization Model (RMM), a mathematical model (Equation (1)) that predicts the proportion of producers (P) in a group based on the amount of food a producer consumes before a scrounger joins the patch, known as the finder’s share (a), quantity of food in the patch (F), and group size (G) [5,6]. In this model, as the finder’s share decreases, the proportion of producers in the group also declines. Calculating the net gain of the finder’s share, considering the time invested (t) in producing the resource, could explain the effect of increasing production costs on the decrease in the proportion of producing responses (Equation (2)).

(1) $$P=\frac{a}{F}+\frac{1}{G}$$

(2) $$P=\frac{a/t}{F}+\frac{1}{G}$$

Afshar and Giraldeau’s [7] agent-based model (ABM) shows that as the cost of producing increases, the proportion of producers decreases. This prediction is consistent with results reported in studies with humans [8] and birds, specifically spice finches (*Lonchura punctulata*) [9] and Carib grackles (*Quiscalus lugubris*) [10]. Results also indicated a positive correlation between the richness of patch zones and the proportion of scroungers, consistent with previous findings by Vickery et al. [4], Beauchamp and Giraldeau [11], and Beauchamp [12].

A substantial body of research on the P-S Game has focused on examining the effects of ecological variables predicted by the Rate Maximization Model (RMM), such as group size and patch richness, in non-human participants. However, in recent years, growing attention has been directed toward understanding the relationship between personality traits and foraging strategies. For instance, individual differences, such as behavioral plasticity [13], which indicates the ability of an individual to adjust their behavior to match or avoid another group’s niche [14], and exploratory tendencies [15], measured by the number of movements an individual makes during its search for resources [15], have been investigated as potential predictors of variation in birds’ social foraging behavior. To our knowledge, there are not many studies exploring the relationship between personality traits and performance in the P-S Game in human participants, even though there is evidence supporting a potential association between these variables in animal behavior [13,15].

Personality is a key element for understanding the adaptive nature of animal behavior. From an evolutionary perspective, it can be defined as consistent individual differences in behavior over time, determined by both genetic factors and environmental influences. These differences arise from phenotypic interactions between socio-ecological contexts and the individual’s developmental and behavioral mechanisms [16]. The study of human personality has focused on the search for the most important individual differences in social interactions, for which there have been a multitude of factorial analyses and lexical approaches over the years to reduce the dimensions and redundant terms observed in different societies, resulting in fewer factor models, commonly five or six, that more effectively describe the variance found throughout different populations [17].

The Big Five model of personality describes the covariation between the cognitive, affective, and motivational traits of an individual through five factors [17]: (1) extraversion, characterized by sociability, assertiveness, and energy, which reflects an energetic approach to their environment, (2) agreeableness, distinguished by trust, compassion, and respect, which describes the use of prosocial and communal behavior even towards antagonistic or hostile individuals, (3) conscientiousness, which exhibits productivity and organization while showing a socially imposed impulse control in order to facilitate behavior towards goal achievement, (4) neuroticism, better represented by anxiety, volatility, and depression, which describes negative emotionality towards concepts such as emotional stability, satisfaction, and frustration tolerance, and, finally, (5) openness, identified by originality and open mindedness, which refers to the depth and complexity of the experiential life of an individual [18].

Despite being initially conceptualized as independent factors, the Big Five personality traits are often correlated between themselves and grouped into two dimensions, stability and plasticity, opening the possibility of a hierarchy inside of the model [19,20,21,22]. Stability, composed of the shared variance of the conscientiousness, agreeableness, and emotional stability (inverse neuroticism) traits, reflects the individual’s ability to maintain relationships and stable emotional states [21], as well as their tendency to evade emotional, motivational, and social disruptions [19]. Plasticity, composed of the shared variance of the extraversion and openness traits, represents a tendency for exploration as well as the level of cognitive and behavioral flexibility shown by an individual when approaching situations in their environment [19,21].

Psychopathy is a multifaceted construct composed of a constellation of deviant personality traits such as a callous–unemotional indifference toward others, affective, interpersonal, and behavioral dysfunctions, and impulsive behavior that leads individuals to disregard social norms without guilt or remorse [23,24,25]. These individuals are unemotional or show very little affect, are completely rational, and consistently act in their own self-interest, disregarding any consequences their behavior may have for others [26].

Neurological evidence points to psychopathy having an influence in different aspects of task performance; for instance, it is related to reduced dorsal Anterior Cingulate Cortex (dACC) activity during tasks, negatively affecting the individual’s capability to link the context of a task with their choice of strategy, which seems to be due to the salience network (SN) failing to deactivate the default mode network (DMN), thus producing competition between attentional resources while engaging in an externally oriented task [27]. Additionally, psychopathic subjects show decreased amygdalar activity when exposed to situations they deem unfair, potentially indicating an attenuation of reactive aggression that allows them to adapt in order to obtain a personal benefit [28].

The measurement of psychopathic personality traits has undergone a significant transition from structured interview methods to self-reporting instruments, largely due to the latter’s convenience in terms of time efficiency for both administration and examiner training [29]. Among these instruments, the Antisocial Process Screening Device (APSD), developed by Frick and Hare [30], stands out as a widely used measure of psychopathic traits. The APSD assesses these traits using two- or three-dimensional structures: callous–unemotional traits, impulsivity, and narcissism, which differ primarily in how the latter two dimensions are distinguished [31,32]. The callous–unemotional factor, in particular, has strong theoretical foundations independent of other facets of psychopathic personality, due to its clinical utility in identifying a subgroup of youths with especially severe antisocial behaviors [31,33].

This study aimed to evaluate the correlation between the choice of social foraging strategies (producing or scrounging), the personality traits of the Big Five model, their metatraits, and the psychopathy traits measured by the Antisocial Process Screening Device (APSD). Additionally, the study pursued the aims of (a) evaluating the effect of the producer response cost and comparing the results with the predictions of Afshar and Giraldeau’s [7] ABM and the RMM [4]; and (b) assessing whether the correlation between the social foraging strategies employed and personality traits remains consistent regardless of increases in the producer response cost.

We hypothesized that traits related to prosocial behavior such as agreeableness and openness correlate positively with producer behavior in a foraging situation, since these individuals are more prone to work in favor of the group and, thus, they might decide to produce resources for the group rather than scrounging them for themselves [18]. We also expected both metatraits to correlate positively with producer behavior in situations where the cost of producing is lower, which favors the use of a producer’s strategy [7,8,9,10]. In contrast, APSD factors should be negatively correlated with producing, but positively correlated with scrounging, considering that individuals that exhibit psychopathic traits are more likely to exploit and take advantage of other people to obtain a benefit [26].

## 2. Materials and Methods

### 2.1. Participants

The present study employed a quantitative, experimental, cross-sectional design. A total of 60 participants aged between 17 and 46 years (M = 23.27, SD = 4.98) were recruited from the southern region of the state of Sonora, Mexico. Ten participants were excluded from the final analyses due to incomplete responses on the personality and psychopathy measures. The final sample consisted of 50 participants, of whom 72% (N = 36) identified as female, 26% (N = 13) as male, and 2% (N = 1) as non-binary. As an inclusion criterion, participants were required to have no prior experience with the behavioral task to prevent potential learning effects or performance advantages.

All participants involved provided their informed consent after letting them know that their participation was completely voluntary and anonymous, reminding them they could leave the experiment at any time, and assuring them of their safety from any psychological or physical harm in its duration. In addition, they were informed of there being monetary prizes for the three best scores of the whole experiment. These research protocols were approved by the Sonora Institute of Technology’s Institutional Review Board (ID 84) to guarantee that the study’s design and procedures carefully followed the ethical principles detailed in the Declaration of Helsinki.

### 2.2. Instruments

**Big Five Inventory (BFI).** The BFI [34] is a 44-item self-report scale that measures the Big Five personality traits: openness, conscientiousness, extraversion, agreeableness, and neuroticism. These items are presented as descriptive sentences that are rated on a 5-point Likert scale (1 = Strongly Disagree; 5 = Strongly Agree). For this study, we used a Spanish translation validated on a Mexican sample by Zamorano et al. [35] with an internal consistency of α = 0.72.

**Antisocial Process Screening Device (APSD).** The APSD [30] is a 20-item self-report scale that assesses three different dimensions of psychopathic behavior: callous–unemotional, impulsivity, and narcissism. Each item is rated on a 3-point Likert scale (0 = Not At All True, 1 = Sometimes True, 2 = Definitely True). In this study, we used a Spanish translated version validated on a Mexican sample by Mejía et al. [32] with an internal consistency of α = 0.79.

### 2.3. Behavioral Task

**Figure 1:**
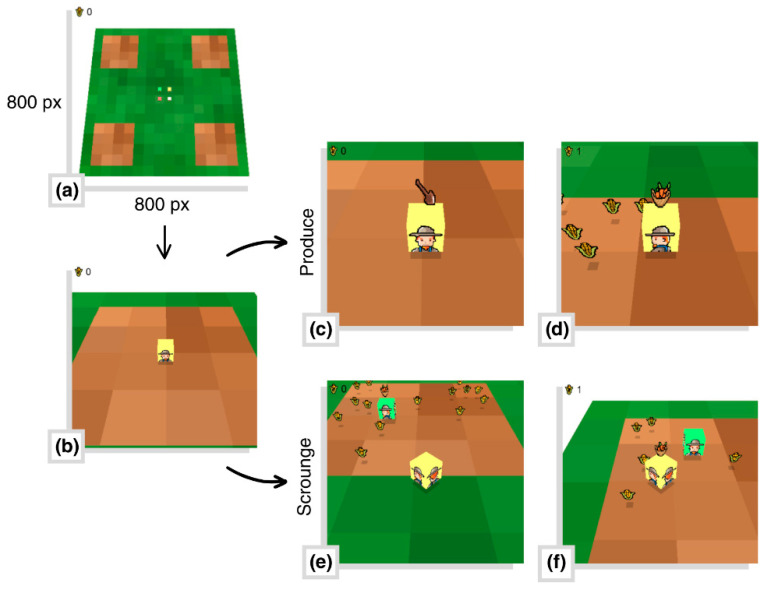
Images of the Guaymas Foraging Task: (**a**) the map where the patch zones and transition zones are located; (**b**) a player inside of a patch zone; (**c**) a producer’s response; (**d**) the player collecting their self-produced food; (**e**) a player joining another player’s discovery; and (**f**) the player collecting the food discovered by someone else (scrounger’s response).

**Guaymas Foraging Task (GFT).** The GFT [6,36] is a direct interaction task in the form of a computer program that allows for the control of the ecological variables of a simulated social foraging episode in which 4 players participate inside a 800 × 800 px virtual 3D environment. This environment is composed of four 200 × 200 px brown-colored patch zones distributed in the corners of the map at an equal distance from each other, while the rest of the space was filled with green transition zones (Figure 1a). Inside of the task, players are able to move freely through the virtual space (Figure 1b) with the objective of collecting the most amount of food units possible. To do this, they can opt to use one of two strategies: (a) producing, which requires the player to press a button for a certain amount of time inside of a patch zone (Figure 1c) in order to find food and ultimately collect it (Figure 1d), or (b) scrounge, which consists of the player joining another group member’s discovery inside of a patch zone (Figure 1e) and collecting part of those resources (Figure 1f).

### 2.4. Procedure

Participants were divided into 15 groups of 4 people, with each group being scheduled for a separate session, taking into account their time availability. Every session took place inside of a 4 × 4 m office inside of the facilities of the Sonora Institute of Technology, in which they were shown the behavioral task on a TV screen that projected the image provided by a laptop equipped with the GFT only accessible to the experimenter. The participants were given a rectangular USB controller to interact with the task. The controller has a directional pad that can be used to move the player character, as well as two buttons on the other side of the controller, even though only the upper one has functionality inside of the game, which is searching for food.

Participants were exposed to two different conditions presented to them in a random order, each one with a duration of 4 min: F5-0 s and F5-8 s. Both conditions were named after two ecological variables: the amount of food units that appear after a successful search (expressed within the first half of the condition’s name with the letter F), and the cost of searching (expressed in the second half in seconds). In the F5-0 s condition, searching took 0.01 s, while in the F5-8 s condition, it took 8 s. In both of them, each search had a 50% chance of success in making the food units appear within the patch zone. Participants were allowed and encouraged to communicate with each other during the experiment.

Prior to the first condition, participants were presented with a pre-training session, in which they were placed in separate spaces with a single patch zone, meant to allow the participants to familiarize themselves with the controls and the mechanics of how to produce and collect food units. The cost of production was set to be the same as the starting condition, but they were only given one food unit after a successful search. This brief pre-training ended after every participant had collected five food units.

After the experiment, participants were instructed to fill out a Google Form that contained sociodemographic data, as well as the BFI and APSD measures. Because of the participants’ personal time restraints, they were asked to answer the form in their free time.

### 2.5. Data Analysis

For this study, we used the producer’s index made by Harten et al. [37] to describe the individual’s foraging behavior during the task. The producer’s index can take values between 1 and −1, where individuals that produce more obtain scores closer to 1, while the ones that scrounge more obtain scores closer to −1 (Equation (3)):

(3) $$Producer’s\;Index\;=\;\frac{Producer’s\;responses\;-\;Scrounger’s\;responses}{Producer’s\;responses\;+\;Scrounger’s\;responses}$$

For the analysis of the Big Five personality traits, we used their average scores rather than their natural scores for every trait, including the metatraits. To measure the stability metatrait, we averaged each participant’s conscientiousness, agreeableness, and emotional stability (inverse neuroticism) scores [19]. To measure the plasticity metatrait, we averaged each participant’s extraversion and openness scores [19]. We used the Shapiro–Wilk normality test to determine the appropriate statistical tests for the comparative analysis between conditions, for which we employed a Wilcoxon signed-rank test. Finally, we used the Pearson correlation coefficient to measure relations between the producer’s index and the Big Five factors of personality, the metatraits, and the APSD factors, as well as the relationship between the factors of both self-report measures.

We used the 12-point cutoff score for the APSD suggested by Pechorro et al. [38] to identify signs of severe antisocial behavior. The comparative analysis was performed using a Mann–Whitney U test after previously testing for normality with the Shapiro–Wilk test and finding the data to be nonparametric.

Seventeen datasets were excluded from the analysis owing to file-saving errors in the GFT. The Appendix A includes the datasets analyzed in this study, the scripts used for data processing and statistical analysis, as well as the executable version of the behavioral task (Guaymas Foraging Task, GFT) available for download.

## 3. Results

**Figure 2:**
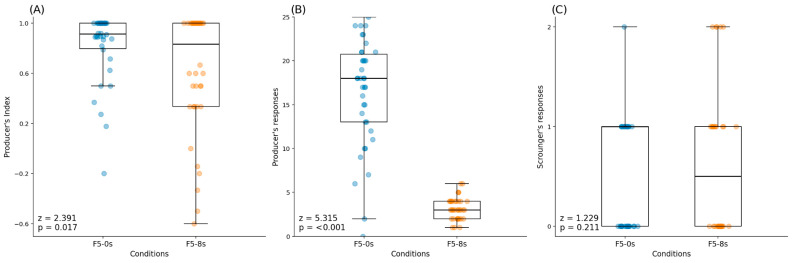
Wilcoxon signed-rank test’s results between conditions F5-0 s and F5-8 s: (**A**) producer’s index between conditions; (**B**) producer’s responses between conditions; and (**C**) scrounger’s responses between conditions.

The comparative analysis showed statistically significant differences in the producer’s index (z (38) = 2.391, *p* = 0.017, rpb = 0.517) and the number of producer’s responses (z (38) = 5.315, *p* < 0.001, rpb = 0.989) between conditions. Both variables had a decrease in their medians; in the case of the producer’s index there was a decrease from 0.920 in F5-0 s to 0.833 in F5-8 s (Table 1, Figure 2A), while the producer’s responses decreased from 18 in F5-0 s to 3 in F5-8 s (Table 1, Figure 2B). There were no statistically significant differences shown in the scrounger’s responses (z (38) = 1.229, *p* = 0.211, rpb = 0.229) (Table 1, Figure 2C).

**Figure 3:**
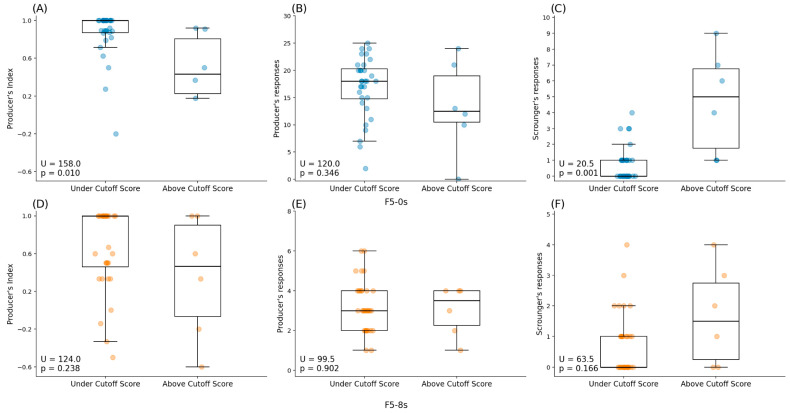
Mann–Whitney U Test’s results between participants under and above the APSD’s cutoff score: (**A**) producer’s index comparison between groups in F5-0 s; (**B**) producer’s responses comparison between groups in F5-0 s; (**C**) scrounger’s responses comparison between groups in F5-0 s; (**D**) producer’s index comparison between groups in F5-8 s; (**E**) producer’s responses comparison between groups in F5-8 s; and (**F**) scrounger’s responses comparison between groups in F5-8 s.

The results showed statistically significant differences between participants under and above the APSD’s 12-point cutoff score in their producer’s index (Table 2, U (38) = 158, *p* = 0.010) and scrounger’s responses (Table 2, U (38) = 20.5, *p* = 0.001) in F5-0 s. The median for the producer’s index was lower in participants above the cutoff score (MDN = 0.434) than in those under (MDN = 1) (Table 2, Figure 3A), while the median for the scrounger’s responses was higher for those above the cutoff score (MDN = 5) than for those under the cutoff score (MDN = 0) (Table 2, Figure 3C). There were not significant differences between groups regarding the producer’s responses (Table 2, U (38) = 120, *p* = 0.238) in F5-0 s, as well as the producer’s index (Table 2, U (38) = 124, *p* = 0.238), producer’s responses (Table 2, U (38) = 99.5, *p* = 0.902), and scrounger responses (Table 2, U (38) = 63.5, *p* = 0.166) in F5-8 s.

**Figure 4:**
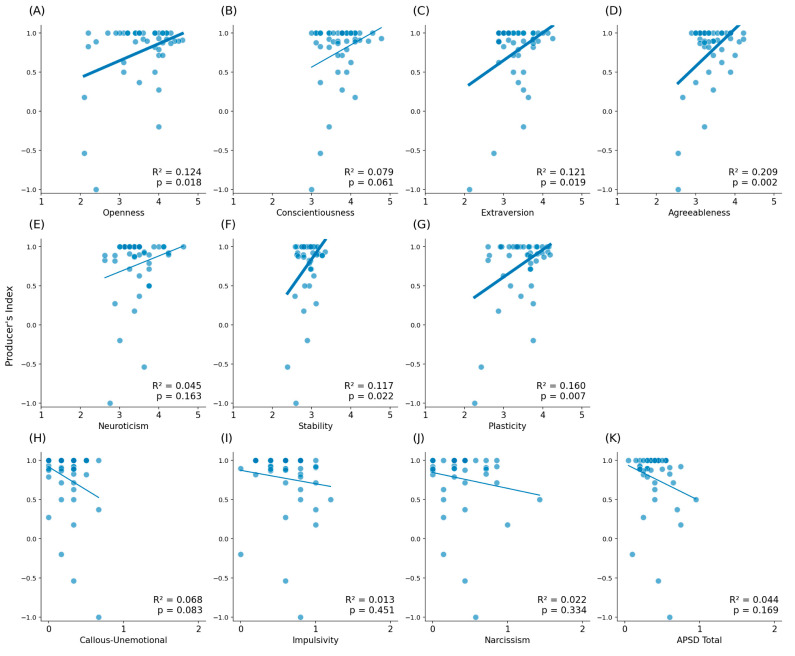
Correlation matrix between the Big Five traits, metatraits, and the APSD with the producer’s index in F5-0 s. Each subsection represents a variable; (**A**–**E**) are the five personality traits, (**F**,**G**) are the metatraits, and (**H**–**K**) are the APSD’s factors. Thicker linear functions represent statistically significant differences.

**Figure 5:**
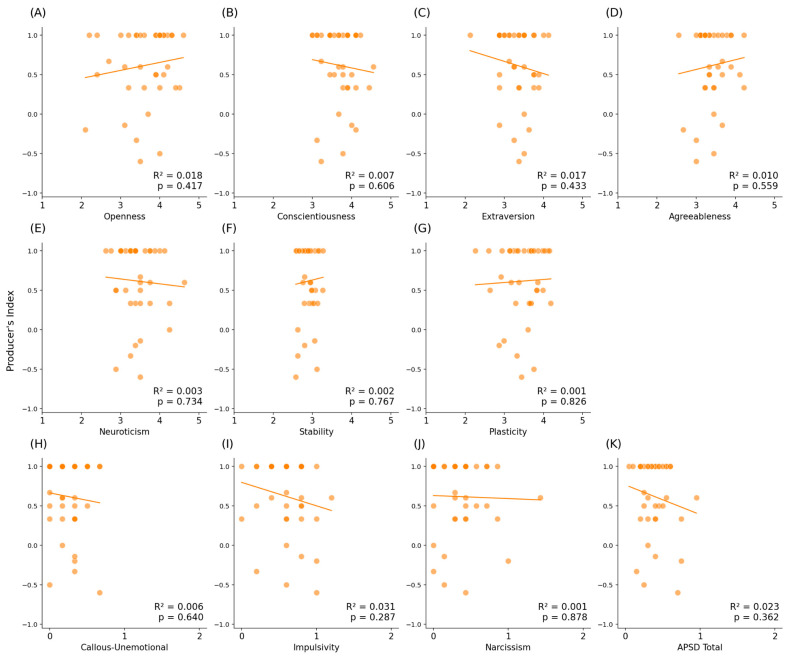
Correlation matrix between the Big Five traits, metatraits, and the APSD with the producer’s index in F5-8 s. Each subsection represents a variable; (**A**–**E**) are the five personality traits, (**F**,**G**) are the metatraits, and (**H**–**K**) are the APSD’s factors.

The results of the F5-0 s condition’s regression analysis regarding the Big Five traits showed statistically significant correlations between the producer’s index and openness (Table 3, Figure 4A, *p* = 0.018, R^2^ = 0.124), extraversion (Table 3, Figure 4C, *p* = 0.019, R^2^ = 0.121), and agreeableness (Table 3, Figure 4D, *p* = 0.002, R^2^ = 0.209). With regard to the metatraits or high-order factors of the Big Five traits, there were statistically significant correlations between the producer’s index and stability (Table 3, Figure 4F, *p* = 0.022, R^2^ = 0.117), as well plasticity (Table 3, Figure 4G, *p* = 0.007, R^2^ = 0.160). However, we did not find any statistically significant correlations between psychopathic traits and the producer’s index (Table 3, Figure 4). There were no statistically significant correlations found between the producer’s index and the five personality traits, metatraits, nor psychopathy in the F5-8 s condition (Table 3, Figure 5).

Finally, there were statistically significant correlations between the conscientiousness trait and the callous–unemotional (Table 3, *p* = 0.001, R^2^ = −0.440) and impulsivity (Table 3, *p* = 0.036, R^2^ = 0.297) factors of the APSD, the agreeableness trait, and narcissism (Table 3, *p* = 0.026, R^2^ = 0.314); and between plasticity and the callous–unemotional factor (Table 3, *p* = 0.040, R^2^ = −0.292).

## 4. Discussion

The present study examined whether individual differences in personality are associated with the use of producing and scrounging strategies in a human social foraging task, and whether such associations persist under different ecological constraints. Drawing on the Producer-Scrounger framework and previous findings in non-human species, we hypothesized that prosocial and exploratory traits, as well as higher-order metatraits related to behavioral flexibility and stability, would be associated with producer behavior, particularly when the cost of producing is low. In contrast, psychopathic traits were expected to relate to increased scrounging. Overall, the results partially supported these hypotheses and, importantly, indicate that the expression of personality-related differences in social foraging is contingent on environmental affordances.

### 4.1. Behavioral Task

The predictions made by the RMM, adjusted to take into account the time invested in producing (Equation (2)), and the ABM made by Afshar and Giraldeau [7] state that as the cost of producing increases, the proportion of producers decreases. The results of this study match with these models’ predictions, showing that the median of both the producer’s index and the producer’s responses were significantly lower in the F5-8 s condition than in the F5-0 s condition. Additionally, these findings are consistent with studies conducted on non-human participants, where this phenomenon can be observed [9,10]. These results suggest that the GFT is a valid task for measuring the strategies used by individuals during a social foraging situation.

### 4.2. The Big Five Traits

The experiment’s results showed positive, statistically significant correlations between the producer’s index and many personality traits, specifically openness, agreeableness, and extraversion, as well as both metatraits: stability and plasticity. Nevertheless, said correlations only appeared in the F5-0 s condition but not the F5-8 s condition, suggesting that personality correlates better with the strategy chosen by an individual inside of a social foraging situation when the cost of producing is lower. This could be because a high production cost decreases the individual’s opportunities to either produce or scrounge, since the more time they invest into searching for resources, the less available said resources become, especially considering that there is a 50% chance that said effort ends in a failed search. This means that they ultimately have fewer chances to express their preferred strategy, which can be backed up with the data obtained in this study showing that the absolute frequency of the producer’s responses changes way more than the absolute frequency of the scrounger’s responses between conditions. That is to say, if high openness, agreeableness, extraversion, stability, and plasticity scores are related to higher producer’s indexes, the influence of personality decreases when the environment, in this case dictated by the condition, limits the individual’s chance of producing by giving them fewer occasions to make decisions during social foraging.

The strongest correlation between the producer’s index and the Big Five traits was found with agreeableness, a trait characterized by a tendency for cooperation, prosocial behavior, and altruism [18,39], which would explain its association with higher producer’s indexes since these individuals could be more prone to produce for the benefit of the group. The trait with the second-highest correlation is openness, described as individual differences in cognitive exploration [39], the willingness to consider new ideas, creativity, and the tendency to search, understand, and utilize sensory and abstract information [39,40], factors that may reflect the individual’s initiative to explore new strategies throughout the search process to find the most effective one, that being producing in the case of a condition with a low production cost. This concept is similar to behavioral flexibility as it is studied in birds [14], a variable that has been shown to have an influence on the use of alternative strategies inside of social foraging [13]. Finally, the last correlation in the five-factor model is extraversion, related to behavioral exploration, sociability, positive emotions, gregarious behavior, and assertiveness [19,39,40], qualities that could allow these individuals to perform better inside of social situations such as the P-S Game, choosing to be cooperative and producing in order to contribute to the group.

### 4.3. Metatraits

Additionally, we found statistically significant correlations between the producer’s index and both of the Big Five model’s metatraits. The strongest association was with plasticity, a metatrait that reflects the person’s flexibility in how they engage with their environment when presented with novel situations [19,21]; taking into account that plasticity has been shown to have an influence on non-human participant decision-making during social foraging [13], this association may extend to human decision-making. The cybernetic function attributed to plasticity as a metatrait by DeYoung [40] relates it to exploration and the creation of new goals, strategies, and interpretations, all of which are core abilities in the P-S Game, allowing these individuals to adapt to the ecological conditions presented to them. Our findings also showed a statistically significant correlation with stability, which reflects a person’s ability and tendency to stay stable and avoid deregulations in emotional, social, and motivational domains [19,20,21]. As with plasticity, it is important to mention its cybernetic function, given by DeYoung [40], that being protecting interpretations, goals, and strategies from being altered by impulses, a function that could translate into the maintenance of a functioning strategy despite situations perceived to be adverse by the individual.

### 4.4. Psychopathy

In contrast to the Big Five model and its metatraits, the callous–unemotional, impulsivity, and narcissism psychopathic traits measured by the APSD did not show any statistically significant correlations with the producer’s index, even though they consistently associated negatively over both conditions. This suggests that, at the dimensional level, these traits may not directly map onto variability in social foraging strategies.

However, a different pattern emerged when participants were categorized using the APSD total score cutoff proposed to identify individuals with marked antisocial behavior [38,41]. Participants scoring above this threshold exhibited significantly lower producer indexes and higher frequencies of scrounging responses under the low-cost condition, suggesting that individuals that show these signs of antisocial behavior scrounge more than those who do not. Nonetheless, similar to the correlations found between the producer’s index and the Big Five traits, this distinction only seems to appear when the cost of producing is low. This could be due to the participants under the cutoff score also producing less and scrounging more in the F5-8 s condition, as shown by the overall decrease in their producer’s index, diluting the effect.

This dissociation between dimensional and categorical findings suggests that psychopathic tendencies may be more effectively captured through threshold-based approaches in the context of social foraging. More broadly, these results underscore the moderating role of ecological constraints: when the cost of producing increases, overall producer behavior declines across participants, thereby reducing variability and obscuring individual differences. Under such restrictive conditions, even individuals predisposed toward exploitation appear constrained in their capacity to preferentially adopt scrounging strategies.

We also analyzed the correlation of the Big Five model and its metatraits with APSD’s factors. Regarding the five personality traits, the results match previous studies by showing that conscientiousness and agreeableness correlated significantly with psychopathic personality traits [42,43]. In the case of this study, responsibility had a negative correlation with the callous–unemotional and impulsivity factors, while agreeableness correlated positively with narcissism. In regard to the metatraits, we found a negative correlation between plasticity and the callous–unemotional factor; however, to our knowledge, the association between the metatraits and APSD’s factors has yet to be explored.

### 4.5. Bootstrapping

To further assess the robustness of these associations, a nonparametric bootstrap procedure with 1000 resamples was conducted, with the resulting confidence intervals reported in the Appendix A. Overall, the bootstrap analyses were consistent with the primary correlational findings, indicating that the observed relationships, particularly those between the producer’s index and openness, agreeableness, extraversion, and the metatraits of stability and plasticity under low-cost conditions, were not driven by single observations or sampling artifacts. Although some confidence intervals were relatively wide, reflecting the moderate sample size, the general pattern of effects remained unchanged. This convergence supports the interpretation that personality-related differences in social foraging emerge reliably when ecological conditions permit strategic flexibility, while becoming attenuated as production costs increase.

### 4.6. Limitations

Differences in previous gaming experience between participants may be a reason for bias in the results, since it could have not only influenced their performance throughout the task but also the strategies they chose. In addition, a few participants expressed annoyance or boredom to some extent during the GFT, which might mean that their behavior inside of the task might not have been a real representation of their producing or scrounging tendencies, but rather a consequence of a lack of motivation.

Finally, although bootstrap analyses supported the stability of the main effects, the relatively wide confidence intervals observed for some correlations highlight the need for future studies with larger samples to more precisely estimate the magnitude of personality–strategy associations.

## 5. Conclusions

The findings presented in this study not only serve as empirical evidence for a possible influence of individual differences on social foraging in human participants, they also offer a different perspective regarding the influence of personality in social situations like the P-S Game. Producer and scrounger behaviors in human participants are not reduced only to the search for food, shelter, or partner, but take part in much larger-scale phenomena such as social hierarchies, politics, academic systems, and health disparities [44]. One of the key concepts to understand this relation is exploitation, an important part of the scrounger strategy in the P-S Game, which describes the scrounger as an individual that exploits a producer’s obtained resources [2]. In this regard, Wells [44] provides several examples of how said exploitation takes part inside of societies creating social disparities, beginning with how people of higher socioeconomic levels benefit from the labor of those with less resources, how, because of gender norms, women are traditionally expected to be the ones to contribute the most to the parenting and maintaining of their children in comparison to men, who, after saving these activities’ energetic costs, are able to spend that saved energy on other activities of their choice, and, by far the clearest example, the slavery of people from other races or ethnic origins throughout history. In relation to this, personality offers a unique perspective on how these exploitative tendencies can be tracked up to certain individual traits, or alternatively, like in the case of this study, show which of these traits are less prone to exploitation or more prone to production.

Furthermore, in addition to the opening of new lines of research to comprehend the basis of these inequalities present in human societies, these findings may provide information about which aspects of people’s personality it is important to appeal to when creating campaigns and organizing movements looking for a change oriented towards social and health equity.

## Figures and Tables

**Table 1:** Comparative analysis between conditions F5-0 s and F5-8 s.

	F5-0 s			F5-8 s			z	*p*	rpb
	M	SD	MDN	M	SD	MDN			
Producer’s index	0.766	0.426	0.920	0.615	0.479	0.833	2.391	**0.017**	0.517
Producer’s responses	16.667	6.762	18	3.184	1.270	3	5.315	**<0.001**	0.989
Scrounger’s responses	1.489	2.370	1	0.895	1.158	0.500	1.229	0.211	0.229

Note. *p*-values in bold indicate statistically significant correlations (*p* < 0.05).

**Table 2:** Comparative analysis between participants under and above the APSD’s cutoff score.

	Condition	Under Cutoff Score (N = 32)			Above Cutoff Score (N = 6)			U	*p*
		M	SD	MDN	M	SD	MDN		
Producer’s index	F5-0 s	0.864	0.255	1	0.312	0.708	0.434	158	**0.010**
	F5-8 s	0.663	0.437	1	0.356	0.650	0.467	124	0.238
Producer’s responses	F5-0 s	16.906	5.579	18	13.333	8.527	12.5	120	0.346
	F5-8 s	3.219	1.289	3	3	1.265	3.5	99.5	0.902
Scrounger’s responses	F5-0 s	0.781	1.099	0	4.667	3.266	5	20.5	**0.001**
	F5-8 s	0.750	1.016	0	1.667	1.633	1.5	63.5	0.166

Note. *p*-values in bold indicate statistically significant correlations (*p* < 0.05).

**Table 3:** Correlation matrix.

	1	2	3	4	5	6	7	8	9	10	11	12
1. Producer’s index F5-0 s												
2. Producer’s index F5-8 s	0.151											
3. Openness	**0.352** *	0.136										
4. Conscientiousness	0.281	−0.087	**0.348** *									
5. Extraversion	**0.348** *	−0.131	**0.492** ***	**0.390** **								
6. Agreeableness	**0.457** **	0.098	0.275	**0.335** *	**0.384** **							
7. Neuroticism	0.212	−0.057	**0.303** *	**0.434** **	**0.301** *	**0.373** **						
8. Stability	**0.342** *	0.050	0.201	**0.613** ***	**0.303** *	**0.610** ***	−0.206					
9. Plasticity	**0.400** **	0.037	**0.924** ***	**0.418** **	**0.788** ***	**0.364** **	**0.347** *	0.275				
10. Callous–Unemotional	−0.261	−0.078	−0.265	**−0.440** **	−0.238	−0.136	−0.239	−0.226	**−0.292** *			
11. Impulsivity	−0.115	−0.177	−0.117	**0.297** *	−0.079	0.129	0.152	0.184	−0.117	−0.206		
12. Narcissism	−0.147	−0.026	0.175	0.093	−0.107	**0.314** *	0.236	0.092	−0.171	0.044	**0.520** ***	
13. APSD Total	−0.209	−0.152	−0.238	0.061	−0.144	0.219	0.180	0.050	−0.231	0.276	**0.720** ***	**0.869** ***

Note. *p*-values in bold indicate statistically significant correlations (*p* < 0.05). Asterisks denote significance levels: * *p* < 0.05, ** *p* < 0.01, *** *p* < 0.001.

## Data Availability

The original data presented in the study is openly available at https://docs.google.com/spreadsheets/d/10RApRLCjrP5Xqj1_KKJLZ8yhYrFcPQCnucLTorN_1cE/edit?usp=drive_link (accessed on 24 February 2025).

## Abbreviations

The following abbreviations are used in this manuscript:

P-S	Producer–Scrounger
RMM	Rate Maximization Model
ABM	Agent-based model
APSD	Antisocial Process Screening Device
BFI	Big Five Inventory
GFT	Guaymas Foraging Task

## Appendix A

To evaluate the robustness and stability of the Pearson correlation coefficients reported in the main text, a nonparametric bootstrap procedure was conducted. This analysis was included to assess the precision of the observed associations given the moderate sample size and to ensure that the reported effects were not driven by single observations or sampling variability.

Bootstrap confidence intervals (95%) were estimated using 1000 resamples with replacements for all pairwise correlations between the producer’s index (F5-0 s and F5-8 s), Big Five personality traits, higher-order metatraits, and psychopathic traits assessed with the APSD. The Pearson’s correlation coefficient was recalculated for each resample, and percentile-based confidence intervals were derived from the resulting empirical distributions.

**Table A1:** Correlation matrix with 95% bootstrap confidence intervals (1000 resamples).

	1	2	3	4	5	6	7	8	9	10	11	12
1. Producer’s index F5-0 s												
2. Producer’s index F5-8 s	0.151 [−0.17, 0.70]											
3. Openness	**0.352** * [**−0.09, 0.61**]	0.136 [−0.16, 0.44]										
4. Conscientiousness	0.281 [−0.07, 0.51]	−0.087 [−0.45, 0.24]	**0.348** * [**0.08, 0.56**]									
5. Extraversion	**0.348** * [**−0.18, 0.65**]	−0.131 [−0.37, 0.14]	**0.492** *** [**0.25, 0.69**]	**0.390** ** [**0.09, 0.60**]								
6. Agreeableness	**0.457** ** [**0.06, 0.66**]	0.098 [−0.25, 0.40]	0.275 [−0.10, 0.55]	**0.335** * [**0.06, 0.57**]	**0.384** ** [**0.07, 0.64**]							
7. Neuroticism	0.212 [−0.08, 0.44]	−0.057 [−0.36, 0.22]	**0.303** * [**0.07, 0.52**]	**0.434** ** [**0.16, 0.66**]	**0.301** * [**0.06, 0.52**]	**0.373** ** [**0.12, 0.62**]						
8. Stability	**0.342** * [**−0.05, 0.58**]	0.050 [−0.38, 0.40]	0.201 [−0.17, 0.49]	0.613 *** [0.38, 0.79]	**0.303** * [**−0.07, 0.58**]	**0.610** *** [**0.41, 0.75**]	−0.206 [−0.45, 0.06]					
9. Plasticity	**0.400** ** [**−0.10, 0.67**]	0.037 [−0.22, 0.34]	**0.924** *** [**0.89, 0.95**]	**0.418** ** [**0.16, 0.60**]	**0.788** *** [**0.64, 0.89**]	**0.364** ** [**0.01, 0.64**]	**0.347** * [**0.13, 0.54**]	0.275 [−0.14, 0.57]				
10. Callous–Unemotional	−0.261 [−0.53, 0.12]	−0.078 [−0.43, 0.31]	−0.265 [−0.49, −0.01]	**−0.440** ** [**−0.66, −0.15**]	−0.238 [−0.50, 0.10]	−0.136 [−0.41, 0.15]	−0.239 [−0.48, 0.04]	−0.226 [−0.50, 0.07]	**−0.292** * [**−0.53, 0.01**]			
11. Impulsivity	−0.115 [−0.41, 0.22]	−0.177 [−0.48, 0.15]	−0.117 [−0.38, 0.15]	**0.297** * [**0.01, 0.57**]	−0.079 [−0.31, 0.16]	0.129 [−0.13, 0.37]	0.152 [−0.14, 0.42]	0.184 [−0.02, 0.42]	−0.117 [−0.35, 0.10]	−0.206 [−0.47, 0.07]		
12. Narcissism	−0.147 [−0.35, 0.09]	−0.026 [−0.35, 0.29]	−0.175 [−0.43, 0.11]	0.093 [−0.14, 0.33]	−0.107 [−0.35, 0.12]	**0.314** * [**−0.02, 0.57**]	0.236 [−0.04, 0.49]	0.092 [−0.14, 0.33]	−0.171 [−0.41, 0.07]	0.044 [−0.17, 0.29]	**0.520** *** [**0.28, 0.70**]	
13. APSD Total	−0.209 [−0.47, 0.12]	−0.152 [−0.47, 0.23]	−0.238 [−0.52, 0.07]	0.061 [−0.22, 0.35]	−0.144 [−0.41, 0.09]	0.219 [−0.13, 0.51]	0.180 [−0.09, 0.43]	0.050 [−0.21, 0.33]	−0.231 [−0.48, 0.03]	0.276 [0.05, 0.51]	**0.720** *** [**0.57, 0.83**]	**0.869** *** [**0.77, 0.93**]

Note. *p*-values in bold indicate statistically significant correlations (*p* < 0.05). Asterisks denote significance levels: * *p* < 0.05, ** *p* < 0.01, *** *p* < 0.001.
